# Comparison of medium-weight vs heavy-weight mesh in minimally invasive extraperitoneal ventral hernia repairs

**DOI:** 10.1007/s00464-026-13476-z

**Published:** 2026-10-15

**Authors:** Ayesha Siddiq, Nicolette M. Winder, Xinyan Zheng, Jeffrey Marks, Joshua L. Lyons, Samuel J. Zolin

**Affiliations:** 1 University Hospitals Cleveland Medical Center, 11100 Euclid Ave, Cleveland, OH 44106 USA; 2 Case Western Reserve University, Cleveland, OH USA; 3 Department of Population Health Sciences, Weill Cornell Medicine, New York, NY USA

**Keywords:** Ventral hernia repair, Minimally invasive surgery, Medium-weight mesh, Heavy-weight mesh, Hernia recurrence, Quality of life

## Abstract

**Background:**

Minimally invasive (MIS) extraperitoneal ventral hernia repair (VHR) has become increasingly common, however, limited data exist comparing medium-weight (MW) versus heavyweight (HW) mesh in this setting. We hypothesized that HW mesh in this context would be associated with decreased rates of hernia recurrence at 1 year and beyond compared to MW mesh.

**Methods:**

We conducted a retrospective analysis of prospectively collected data from the Abdominal Core Health Quality Collaborative database, identifying adults who underwent MIS extraperitoneal (preperitoneal and/or retromuscular) VHR with either MW (40–60 g/m^2^) or HW (> 75 g/m^2^) uncoated permanent synthetic mesh from the inception of the registry to May 2025, with ≥1 year clinical or patient-reported follow-up. Propensity score matching was used to adjust for imbalances in baseline patient characteristics. The primary outcome was 1-year pragmatic hernia recurrence, defined as recurrence based on available elements of patient-reported, clinical and/or radiographic recurrence. Secondary outcomes included 30-day surgical site infection (SSI), surgical site occurrences (SSO), surgical site occurrences requiring procedural intervention (SSOPI).

**Results:**

821 patients were identified. After propensity score matching, 238 patients remained (119 MW, 119 HW). Most baseline covariates were well-matched. Patients with umbilical hernias were more likely to receive MW mesh (80% vs. 65%, *p* =0.009), while patients receiving HW mesh were more likely to have subxiphoid hernias (5.9% vs 15%, *p* =0.020). There were no documented SSI at 30 days. The rates of SSO (*p* =0.696), SSOPI (*p* =0.498), and 30-day reoperation (*p* =0.999) were similar between groups. At 1-year, pragmatic recurrence was similar between MW and HW patients (7.6% vs 12.6%, *p* =0.196).

**Conclusions:**

MW and HW mesh demonstrate comparable early and mid-term outcomes following MIS extraperitoneal VHR. Further prospective study and long-term follow-up are necessary as these techniques proliferate.

**Graphical abstract:**

**Figure:**
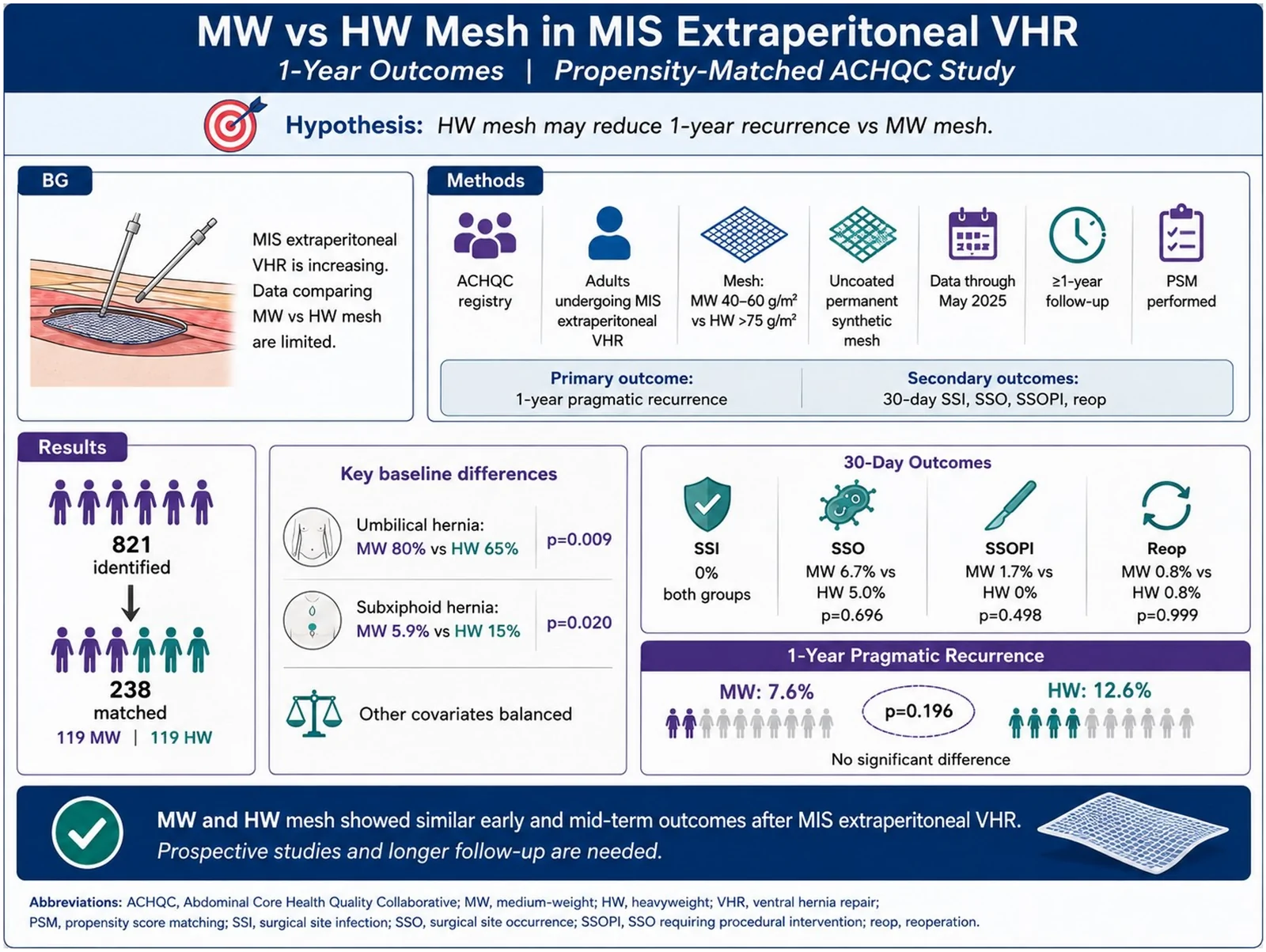

Minimally invasive surgery (MIS) has become an increasingly common approach for ventral hernia repair (VHR) over the past decade. Greater access to robotic platforms as well as increased surgeon experience with this technology have allowed surgeons to undertake operations that previously would have been performed in an open fashion, namely preperitoneal approaches and retromuscular component separation techniques. Likewise, many surgeons have turned toward extraperitoneal approaches regardless of hernia size to avoid intraperitoneal mesh placement. Recent analyses of the Abdominal Core Health Quality Collaborative (ACHQC) data have shown a clear shift toward extraperitoneal repairs in MIS VHR, with preperitoneal and retromuscular approaches increasing from just 10% of surgeon-reported MIS repairs in 2013 to over 50% by 2021 [1].

Consideration of the technical aspects of these operations, including prosthetic selection, should therefore be a research priority. A prior randomized controlled trial (RCT) comparing medium-weight (MW) and heavyweight (HW) mesh in open retromuscular VHR found no significant differences in recurrence at 1 year [2]. Longer-term results from this study have not yet been published. However, other long-term non-randomized data raise concern about potentially higher rates of hernia recurrence with use of MW mesh in a retromuscular position [3]. Even in cases where fascial closure was achieved, there was still a clinically significant 3.3% rate of central mesh fracture in patients undergoing repairs with MW mesh.

Another recent analysis comparing robotic and open retromuscular VHR identified that patients undergoing robotic-assisted retromuscular VHR were at a potentially higher risk of long-term recurrence [4]. The exact mechanism for this is unknown, but differences in mesh selection between open and minimally invasive contexts might be a contributing factor. Despite these findings, no large-scale analysis has investigated whether mesh weight might similarly affect outcomes after MIS extraperitoneal VHR.

In this study, we evaluated early and mid-term outcomes of HW vs MW mesh in the setting of MIS extraperitoneal VHR, including both retromuscular and preperitoneal approaches. We hypothesized that the use of HW mesh would be associated with decreased rates of pragmatic hernia recurrence at 1 year and beyond compared to MW mesh.

## Methods

We conducted a retrospective analysis of prospectively collected data from the ACHQC database. The ACHQC is a national registry designed to facilitate continuous quality improvement and outcomes of research in abdominal-wall reconstruction, featuring data from more than 150,000 patients entered by over 500 participating surgeons [5]. Adult patients (≥ 18 years) who underwent MIS (laparoscopic or robotic), extraperitoneal (retromuscular and/or preperitoneal) VHR from registry’s inception in 2013 through May 2025, with at least 1 year clinical or patient-reported follow-up were included. Eligible cases included only clean wound class and cases in which fascial closure was achieved. Cases with concomitant inguinal or hiatal hernia repair, open repair, MIS conversion to open repair, use of multiple meshes, or missing covariates required for matching were excluded.

Only patients with documented mesh type and size were included. All included cases used either MW (40–60 g/m^2^) or HW (> 75 g/m^2^) uncoated permanent synthetic mesh. Cases with missing mesh characteristics were excluded. Relevant past medical and surgical history, technical aspects of the operation, and postoperative clinical and patient-reported outcomes were recorded.

Hernia recurrence was measured in three ways: (1) surgeon-reported recurrence based on physical exam or imaging, (2) patient-reported recurrence based on a positive response to the Hernia Recurrence Inventory (HRI), which is a validated screening tool for detection of hernia recurrence, and (3) pragmatic recurrence, which is a composite measure of available surgeon-reported and patient-reported recurrence measures at a given time point, allowing integration of discordant or incomplete measures of recurrence to provide a “best available” measure of recurrence [6]. For instance, a patient with a positive response to the HRI and no clinical follow-up would be considered to have a pragmatic recurrence. However, if this same patient were seen by a surgeon and determined not to have a clinical recurrence, they would be considered not to have a pragmatic recurrence. The primary outcome was pragmatic hernia recurrence at 1 year, as defined in a previous RCT, though all contributing measures of recurrence are reported in an effort to maximize transparency in light of imperfect registry follow-up [2]. Secondary outcomes included 30-day surgical site infection (SSI) or surgical site occurrences (SSO), SSI or SSO requiring procedural intervention (SSOPI), 30-day readmission, reoperation within 30 days, and recurrence at 30 days. Patient-reported outcomes were also assessed at baseline, 30 days, and 1 year, including pain as measured by the Patient-Reported Outcomes Measurement Information System (PROMIS) Pain Intensity score and abdominal-wall–specific quality of life using the hernia-specific quality of life (HerQLes) survey [7, 8]. A higher PROMIS score indicates a greater level of pain; the minimum clinically important difference (MCID) was defined as a range from 2.0 to 3.0 points [9]. A higher HerQLes score indicates a better quality of life; with MCID defined as an improvement of at least 15.6 points [10].

To adjust for potential confounding, we used propensity score matching to create comparable groups based on demographic and clinical variables, including age, gender, BMI, diabetes, smoking status, whether a hernia was primary or recurrent, incisional hernia, hernia width, European Hernia Society (EHS) classification of midline hernia, lateral hernia, or both, use and extent of myofascial release, drain usage and mesh sublay placement location (retrorectus/retromuscular and/or preperitoneal) [11]. Propensity scores were estimated using logistic regression and matched in a 1:1 nearest neighbors’ fashion (caliper width was 20% of the standard deviation of the logit of the propensity score). Statistical analysis was performed using R version 4.5.2 (Vienna, Austria).

Medians and interquartile ranges (IQR) were used to summarize continuous variables. Standardized mean differences were used to examine covariate balance after matching. Matched cohorts were then analyzed Chi-squared tests for categorical variables and Wilcoxon rank-sum tests for continuous variables. Time-to-event outcomes were analyzed using Kaplan–Meier methods, with comparisons between groups performed using log-rank test. This was carried to approximately 2 years of follow-up, after which point the rates of follow-up were low and the model became unstable. A *p*-value of <0.05 was considered statistically significant. Statistical analysis was performed by an ACHQC-affiliated statistician (XZ) and no patient-level data were available to the investigators.

Institutional Review Board (IRB) approval was obtained for this study, and all data were collected in compliance with ethical research standards.

## Results

Out of 146,593 patients in the ACHQC database at the time of analysis, 821 met inclusion criteria. After propensity score matching, 238 patients remained in the analytic cohort (119 MW, 119 HW).

Table 1 demonstrates demographic characteristics of the patient cohort. After 1:1 matching, most baseline demographic and hernia-related variables were well balanced between both groups however, several differences persisted (Table 1, 2). Regarding operative characteristics, the MW group had a larger median mesh length (25 [15–30] cm vs. 22 [16–30] cm, *p* =0.025), while mesh width was statistically similar (Table 3). Patients receiving MW mesh were more likely to have M3 (umbilical) hernias (80% vs 65%, *p* =0.009), whereas patients receiving HW mesh were more likely to have M1 (subxiphoid) hernias (5.9% vs 15%, *p* =0.020) (Tables 1 and 2). Regarding operative characteristics, the MW group had a larger median mesh length (25 [15–30] cm vs. 22 [16–30] cm, *p* =0.025), while mesh width was statistically similar (Table 3).

**Table 1:** Patient demographic characteristics after propensity score matching^a^

Demographics	MW (*n =*119)	HW (*n =*119)	*p*-value
Age, years	63 (53–69)	63 (52–70)	0.894
BMI, kg/m^2^	34 (29–40)	32 (29–37)	0.382
Sex			0.299
Female	60 (50)	52 (44)	
Male	59 (50)	67 (56)	
Comorbidities			
Diabetes mellitus	26 (22)	22 (18)	0.518
COPD	7 (5.9)	5 (4.2)	0.554
Smoking(current)	7 (5.9)	10 (8.4)	0.450
Immunosuppression	5 (4.2)	3 (2.5)	0.722
ASA class			0.325
1	3 (2.5)	3 (2.5)	
2	46 (39)	58 (49)	
3	70 (59)	58 (49)	

^a^Data are presented as median (IQR) or n (%)

**Table 2:** Hernia-specific history^a^

Hernia Characteristics	MW (*n =*119)	HW (*n =*119)	*p*-value
Hernia type			0.288
Incisional	103 (87)	97 (82)	
Primary	16 (13)	22 (18)	
Hernia length, cm	10.0 (5.0–15.0)	8.0 (5.0–15.0)	0.411
Hernia width, cm	5.0 (4.0–8.0)	5.0 (4.0–8.0)	0.799
Recurrent hernia	34 (29)	24 (20)	0.131
Midline hernia	99 (83)	96 (81)	0.857
M1–Subxiphoidal	7 (5.9)	18 (15)	0.02
M2–Epigastric	63 (53)	65 (55)	0.795
M3–Umbilical	95 (80)	77 (65)	0.009
M4–Infraumbilical	44 (37)	44 (37)	>0.999
M5–Suprapubic	10 (8.4)	11 (9.2)	0.819
No Midline component	17 (14)	19 (16)	0.717
Lateral hernia	17 (14)	19 (16)	0.857
L1–Subcostal	2 (1.7)	2 (1.7)	>0.999
L2–Flank	8 (6.7)	9 (7.6)	0.801
L3–Iliac	7 (5.9)	5 (4.2)	0.554
L4–Lumbar	4 (3.4)	11 (9.2)	0.062
No Lateral component	99 (83)	96 (81)	0.613
Both midline and lateral hernia	3 (2.5)	4 (3.4)	0.857

^a^Data are presented as median (IQR) or n (%). Hernia location was classified according to the European Hernia Society (EHS) classification system, with midline (M) and lateral (L) designations used to describe the anatomical location of included hernias

**Table 3:** Operative characteristics^a^

Operative characteristics	MW (*n =*119)	HW (*n =*119)	*p*-value
Operative approach			0.683
Robotic	117 (98)	115 (97)	
Laparoscopic	2 (1.7)	4 (3.4**)**	
Elective Repair	117 (98.3)	116 (97.5)	> 0.99
Preperitoneal sublay placement	36 (30)	40 (34)	0.578
Retrorectus/retromuscular sublay placement	87 (73)	84 (71)	0.665
Mesh length, cm	25 (15–30)	22 (16–30)	0.025
Mesh width, cm	19 (15–25)	18 (15–23)	0.1
Mesh length overlap, cm	7 (5–9.5)	6 (4.5–8)	0.02
Mesh width overlap, cm	6.75 (5–8.5)	6 (4.5–7.5)	0.022
Myofascial release	81 (68)	78 (66)	0.68
Posterior rectus sheath	74 (91)	67 (86)	0.277
Transverse abdominus	41 (51)	31 (40)	0.169
Mesh fixation			
Sutures	52 (75)	51 (75)	0.961
Tacks	7 (10)	7 (10)	0.977
Adhesives	11 (16)	12 (18)	0.789
Operative time			0.953
0–59 min	4 (3.4)	3 (2.5)	
60–119 min	28 (24)	30 (25)	
120–179 min	33 (28)	36 (30)	
180–239 min	28 (24)	28 (24)	
≥240 min	26 (22)	22 (18)	

^a^Data are presented as median (IQR) or n (%)

**Fig. 1:**
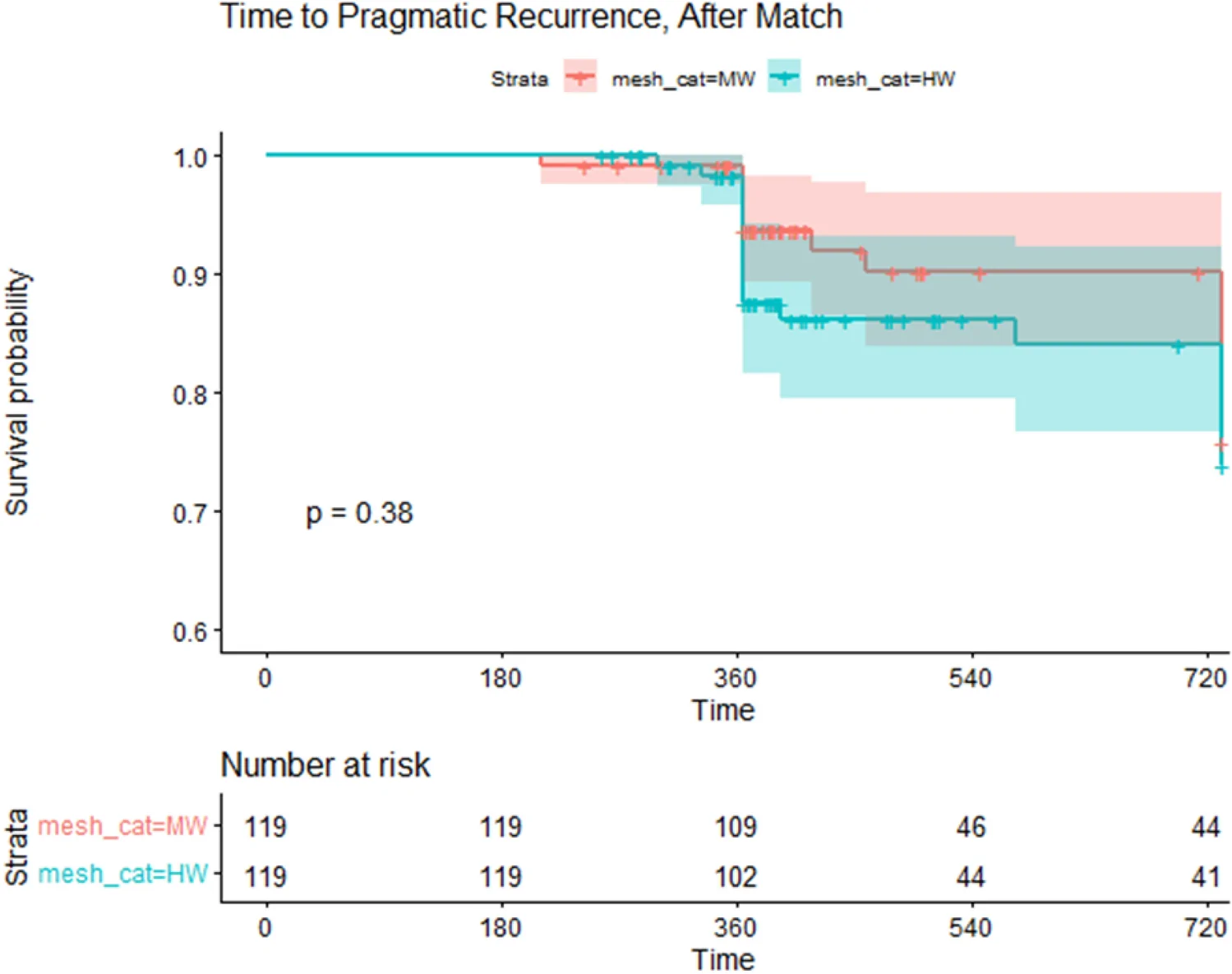
Kaplan Meier curve for time to pragmatic recurrence after propensity score matching. Groups compared using log-rank test

The primary outcome, pragmatic hernia recurrence at 1 year, occurred in 9 MW patients (7.6%) and 15 HW patients (12.6%), a difference that was not statistically significant (*p* =0.196). There was a greater rate of patient-reported bulging at 1 year in the HW group (23% vs 11%, *p* =0.045). Surgeon-reported recurrence at 1 year did not show a statistically significant difference either (*p* =0.729) (Table 4). Kaplan–Meier analysis demonstrated similar recurrence-fee survival between groups, with no significant difference observed (log-rank *p* =0.38) (Fig. 1).

**Table 4:** Rates of recurrence^a^

Recurrence Type	N	MW (*n =*119)	HW (*n =*119)	*p*-value
Surgeon reported				
30 days	238	0 (0.0)	0 (0.0)	0.729
1 year	103	3 (6.5)	5 (8.8)	
Patient reported				
30 days	151	16 (22)	24 (31)	0.218
1 year	180	11 (11)	19 (23)	0.045
Pragmatic recurrence				
1 year	238	9 (7.6)	15 (12.6)	0.196

^a^Data are presented as median (IQR) or n (%)

Rates of SSO at 30 days were comparable between groups (*p* =0.696), and there were no documented SSI at 30 days. SSOPI at 30 days occurred in 2 (1.7%) HW patients and no MW patients (*p* =0.498). Likewise, 30-day readmissions and reoperations within 30 days were rare and did not differ significantly between cohorts (Table 5).

**Table 5:** Post-operative outcomes^a^

Outcome	N	MW (*n =*119)	HW (*n =*119)	*p*-value
At 30 days follow-up				
SSO	238	14 (12)	16 (13)	0.696
SSI	238	0 (0)	0 (0)	>0.999
SSOPI	238	0 (0)	2 (1.7)	0.498
Reoperation	238	0 (0)	1 (0.8)	>0.999
At 1 year Follow-up				
SSO	106	3 (6.1)	5 (8.8)	0.722
SSI	106	0 (0)	1 (1.8)	>0.999
SSOPI	106	1 (2.0)	3 (5.3)	0.622
Reoperation	108	4 (8.0)	4 (6.9)	>0.999

^a^Data are presented as n (%)

Rates of SSO at 1 year were comparable between both groups (*p* =0.722). SSI was rare at 1 year and not statistically significant (*p* > 0.999). SSOPI at 1 year was numerically greater although not statistically significant (5.3% vs 2%, *p* =0.622) (Table 5).

Median HerQLes score at 30 days was slightly higher in the HW group (62 [42–88] vs 55 [33–73], *p* =0.071], showing an increase from baseline in both cohorts (48 [20–68] vs 39 [19–74], *p* =0.507). By 1 year, the median scores improved and were comparable for both groups (91 (75–95) vs 87 [57–100], *p* =0.463) (Table 6).

**Table 6:** Quality-of-life outcomes^a^

Outcomes	N	MW (*n =*119)	HW (*n =*119)	*p*-value
HerQLes Score				
Baseline	110	39 (19–74)	48 (20–68)	0.507
30 days	153	55 (33–73)	62 (42–88)	0.071
1 year	170	87 (57–100)	91 (75–95)	0.463
PROMIS pain T score				
Baseline	111	49 (40–55)	44 (36–52)	0.16
30 days	155	46 (44–52)	45 (40–49)	0.24
1 year	173	36 (31–46)	31 (31–40)	0.061

^a^Data are presented as median (IQR)

Similarly, the median PROMIS score at 30 days did not show a significant difference between both groups (46 [44–52] vs 45 [40–49], *p* =0.240) or at baseline (49 [40–55] vs 44 [36–52], *p* =0.160). At 1 year the median PROMIS score was again comparable for both groups (36 [31–46] vs 31 [31–40], *p* =0.061) but demonstrated an overall decrease from 30 days (Table 6).

## Discussion

To our knowledge, this is the first investigation of the impact of mesh weight on clinical and patient-reported outcomes of extraperitoneal MIS VHR. At 1 year, pragmatic hernia recurrence rates were similar between groups, though there was a significantly higher rate of patient-reported bulging in the group receiving HW mesh. Rates of other short- and long-term outcomes including SSO, SSI, SSOPI, and the need for reintervention were also comparable. These findings suggest that mesh weight alone may not be a primary determinant of recurrence or postoperative complications in this operative context.

The topic of mesh weight as it relates to clinical outcomes after open VHR has gained increased attention over the last several years, with two recent meta-analyses investigating this topic [12, 13]. In both meta-analyses, HW mesh was found to confer an increased likelihood of foreign body sensation compared to non-HW mesh, but without significant differences in other clinical outcomes such as hernia recurrence, wound infection, or chronic pain. When restricted to the four RCTs that have investigated HW mesh compared to non-HW mesh, again, no difference was found with regard to recurrence, wound infection, or postoperative pain [2, 14–16]. Of note, the present study utilizes the same mesh density cutoffs as definitions of MW and HW and additionally included only patients who were able to achieve fascial closure, mirroring the inclusion criteria of the RCT conducted by Krpata et al., given that that study was also based in the ACHQC and utilized many of the same outcome measures [2]. Our findings are consistent with these prior comparative studies demonstrating no clear association between mesh weight and hernia recurrence in the extraperitoneal space.

A subset of recurrences following MW mesh repair have been attributed to central mesh fractures, particularly in larger hernias repaired with bridged fascial closure. In the most extensive series on MW mesh fractures, Maskal et al. describes a cohort of patients with mesh fracture whose median hernia width was 20 cm. In the present study, the median hernia width was 5 cm [3]. It is likely that for the present cohort, fascial closure was able to occur under significantly less tension, and therefore the prosthetic used was less impactful. Further prospective investigation in the context of patients undergoing robotic transversus abdominis release (TAR) for larger defects is needed.

One possible explanation for the observed similarity in outcomes is that VHR success is driven primarily by surgical technique rather than mesh material characteristics alone. Adequate fascial closure and sufficient mesh overlap may be more essential than the weight of mesh used [17]. Slight differences in mesh overlap between groups in the present study may reflect technical and material-specific factors. Heavyweight mesh is inherently less pliable and may be more difficult to manipulate in minimally invasive operations. As a result, surgeons may preferentially select smaller mesh sizes to facilitate intracorporeal handling and positioning. Additionally, there may be concern that excess heavyweight mesh within a confined extraperitoneal pocket could predispose to folding or wrinkling at the edges, potentially impairing mesh incorporation. In contrast, medium-weight mesh, due to its greater flexibility and lower material density, may allow for easier deployment of larger prostheses. These differences in intraoperative handling and sizing may partially explain the greater overlap observed in the MW cohort and could contribute to the numerically higher recurrence rates seen in the HW group.

With regard to the higher rate of patient-reported bulging observed in the HW group, a greater proportion of patients in the HW group had M1 (subxiphoid) defects, which may create more difficulty closing fascia related to adjacent ribs acting as a fixed bony structure. Further, a greater, though not statistically significant, number of patients receiving HW mesh in the current study had lumbar hernias. It is possible that the combination of these two populations predisposed to a greater rate of patient-reported bulging. Additionally, it is plausible that in response to the HRI question “do you see or feel a bulge,” some patients may have responded positively to indicate a foreign body sensation, consistent with the previously noted higher rate of foreign body sensation with HW mesh seen in existing literature.

In all likelihood, our study is underpowered to detect a significant difference in hernia recurrence rates between groups. Pragmatic recurrence rates of 7.6% and 12.6% in the MW and HW groups respectively would be considered clinically significant by most surgeons, even if not statistically significant in the present analysis. Importantly, our study provides these as recurrence estimates which may be pooled in future meta-analyses and which would help in designing future randomized controlled trials. Utilizing these rates of hernia recurrence, power of 90%, *p* =0.05, and accounting for 10% loss to follow-up, approximately 1660 patients would be needed for such a trial. This would obviously be a massive undertaking. Fewer than half of this number had long-term follow-up in the pre-match cohort, and these operations were performed by surgeons highly invested in following their own outcomes. These results highlight the critical need for surgeon and patient engagement in long-term follow-up and collaborative data sharing through registry participation as robotic extraperitoneal hernia repair techniques become ever more popular.

In addition to sample size, as noted above, our study has several other limitations. The retrospective design can be a source of unmeasured confounding. We have attempted to mitigate this through use of propensity score matching, adjusting for factors that we felt were likely to influence outcomes. Despite this, it is possible that other unknown or unmeasured confounders might exist. We limited our cohort to patients who had at least 1 year follow-up. It is possible that patients who experienced an adverse outcome, including hernia recurrences, may have not followed up with their original surgeon and may not be represented in this data. Unfortunately, there is no way to account for this at present. Patient-reported bulging, while clinically meaningful to patients, is a subjective measure of recurrence and may be overly sensitive for the detection of hernia recurrence. Furthermore, follow-up beyond one year continued to drop off, and we know that hernia recurrence rates increase with time [18]. Despite these limitations, this is the most complete and granular data available at this time.

In the context of MIS extraperitoneal VHR, there does not seem to be a significant difference in long-term performance of MW versus HW mesh. Technical factors including adequate fascial closure and mesh overlap likely have greater roles in hernia recurrence outcomes. Additional observational follow-up and ideally prospective, randomized studies with long-term follow-up are needed to determine whether mesh weight may influence late recurrence, chronic pain, or other quality-of-life outcome in this operative context. These outcomes, in addition to economic analysis, may further clarify the role of mesh characteristics in optimizing hernia repair outcomes.
